# Time influence in the enzymatic saccharification of cellulose pulp samples

**DOI:** 10.1186/1753-6561-5-S7-P112

**Published:** 2011-09-13

**Authors:** Cristiane V Helm, Washington L E Magalhães, Edson A de Lima, Patrícia R Silva, Kleber Hoffmann, Amanda Higa, Dayanne Mendes

**Affiliations:** 1 Embrapa Forestry, Brazilian Agricultural Research Corporation (Embrapa), Colombo, Paraná, 83411-000, Brazil; 2 Federal University of Paraná, Curitiba, Paraná, 80060-000, Brazil

## Background

With growing concern in obtaining clean and renewable energy, many works in bioenergy has been developed in order to obtain substantial gains for the environment.

Due to the large environmental impact associated to the use of fossil fuels, ethanol production from lignocellulosic materia lhas been widely explored, aiming environmental, economic and social benefits.

Lignocellulosic biomass, composed primarily of cellulose, hemicellulose, and lignin, can be submitted to enzymatic hydrolysis in order to produce reducing sugars, which in turn can be converted into ethanol. However, for the whole process to become economically feasible, optimization of the conditions is necessary [1].

To find innovative solutions for the use of lignocellulosic wastes, the aim of this study was to evaluate the ability of converting cellulose bleached pulp into reducing sugars from enzymatic hydrolysis, at different exposure times.

## Methods

Cellulose pulp samples were weighed in the amount of 0.2 g and transferred to Falcon tubes, in which were added 40 mL of 50 mM sodium citrate buffer with pH 4.8. For hydrolysis, 0.05 mL of cellulase (Celluclast 1.5 L) and 0.05 mL of cellobiase (Novozyme 188) enzymes were transferred to each tube. The Falcon tubes were incubated in shaker at 50±1 °C and 250 rpm for 24, 48, and 72 hours. Subsequently, reducing sugars were quantified through the dinitrosalicylic acid (DNS) method [2].

## Results and conclusions

The results were very similar for all incubation times. The average reducing sugar concentrations were 4.15 g/L, 4.14 g/L and 4.16 g/L, for 24, 48, and 72 hours, respectively. The composition of substrate was: 98 % cellulose, 1.7 % humidity and 0.3 % ashes. Cellulose conversion into sugar was calculated considering that cellulose was converted mainly into glucose, in a proportion of 1.11 g glucose/g cellulose. The conversion percentages obtained were: 76.3 %, 76.1 % and 76.5 %, for 24, 48 and 72 hours, respectively. These results show that the hydrolysis incubation time higher than 24 hours does not improve the conversion of cellulose into glucose. One possible explanation would be the fact that the enzymes mainly hydrolyze amorphous or less ordered crystalline cellulose. Therefore, remains just highly crystalline cellulose molecules, hindering the access of the enzyme. In other words, this highly crystalline cellulose prevents the total conversion, even over long periods of time. X-ray diffraction (XDR) measurements will be done to check this hypothesis.
